# Effect of Belladonna upon an Irritable Eye

**Published:** 1841-11

**Authors:** 


*Effect of Belladonna upon an Irritable Eye.*—A female of highly nervous temperament, who had been blind from cataract, and had been the subject of several operations for its cure, applied for advice for a diseased condition of the left eye, which followed the removal of cataract, and formed a serious obstacle to vision. The operation, which was performed upon this eye more than a year since by myself, had been followed by a severe and protracted attack of rheumatic ophthalmia, which resulted in a thickening of a portion of the capsule of the lens which had not been absorbed, and an adhesion to it of the papillary margin of the iris, forming an enlarged and irregular pupil. The lens was probably entirely absorbed, and the thickened capsule was perforated by two orifices, one toward the inner and the other toward the outer canthus, through which the patient could see by directing the eye outward or inward, although she was unable to distinguish objects directly in front of her. In the right eye the iris was moveable, the pupil clear with the exception of a minute portion of the lens which appeared in front, and the sight good, the patient being able to read large print without difficulty. Previous to a critical examination of the eyes, I directed (as is usual) the application of ext. of belladonna to the lids at bed-time, intending to inspect them in the morning of the following day. At this time I found the pupil of the right eye (which was the better of the two) largely dilated, but the patient was complaining that her sight was much worse since the application. She was troubled with muscæ volitantes, and other illusive appearances indicative of partial amaurosis; I trusted that these symptoms would disappear, as the effect of the belladonna went off, but they have continued more or less to the present time, a period of several months, much to the annoyance of the patient, who complains bitterly of the change effected in her sight since this application.

The danger of the free application of belladonna or stramonium in cases where there is the least tendency to amaurosis, has been suggested by some authors; while by others, amongst whom is Lawrence, such fears have been considered groundless. The case cited is the only one in which I have remarked any strikingly injurious effects from it, after having seen it used freely and extensively prior to the operation for cataract. That the powerful atonic effect which this article is capable of producing should, however, promote the tendency to amaurosis, where it exists, appears reasonable, and should induce caution in its use where we have doubts as to the soundness of the retina.—*Amer. Jour. Med. Sci. Oct.* 1841.

